# Targeting the brain-gut-prostate axis in chronic prostatitis: mechanisms and therapeutics

**DOI:** 10.3389/fendo.2025.1628094

**Published:** 2025-07-04

**Authors:** Shiwei Song, Chunlei Zhang, Bin Zhang, Jinlong Yin, Chang Yu, Xuanrong Wang, Qing Wang, Fulin Ma, Changfeng Yang, Dehui Chang

**Affiliations:** ^1^ Department of Urology, 940th Hospital of the Joint Logistics Support Force, Lanzhou, China; ^2^ First School of Clinical Medicine, Gansu University of Chinese Medicine, Lanzhou, China

**Keywords:** chronic prostatitis, gut microbiota, neuroimmune interactions, therapeutics, CP/CPPS

## Abstract

Chronic prostatitis/chronic pelvic pain syndrome (CP/CPPS), a refractory urinary system disorder, is closely associated with dysregulation of the brain-gut-prostate axis. Emerging evidence highlights the pivotal role of gut microbiota dysbiosis and its bidirectional interactions with the neuroimmune system in CP/CPPS pathogenesis. This systematic review integrates perspectives from microbiomics, neuroimmunology, and metabolomics to propose a theoretical framework of the brain-gut-prostate axis and multi-dimensional therapeutic strategies targeting this axis. By transcending conventional localized anti-inflammatory approaches, these strategies aim to address clinical resistance and phenotypic heterogeneity. Mechanistic insights into microbiota-derived metabolites (e.g., short-chain fatty acids, SCFAs), neuroendocrine signaling (e.g., thyrotropin-releasing hormone, TRH), and immune crosstalk (e.g., Th17/Treg imbalance) are explored, alongside innovative therapies such as microbiome modulation, neural interventions, and immune regulation. This holistic paradigm not only provides new mechanistic insights but also offers promising avenues for personalized management and translational research in CP/CPPS, potentially overcoming current therapeutic bottlenecks.

## Graphical Abstract

Gut microbiota dysbiosis disrupts the brain-gut-prostate axis via neuroimmune-metabolite crosstalk, driving chronic prostatitis pain and inflammation. Targeting this axis offers novel therapeutic strategies.

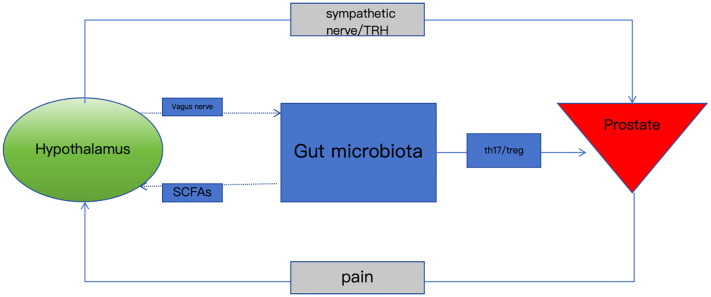

## 1 Introduction: shifting from local prostatic inflammation to systemic regulation

Chronic prostatitis/chronic pelvic pain syndrome (CP/CPPS) affects approximately 8.2% of males globally (1), presenting with persistent pelvic pain, urinary dysfunction, and significant quality-of-life impairment. Its pathogenesis involves complex interactions among neuroinflammation, pelvic floor dysfunction, and immune dysregulation, contributing to high phenotypic heterogeneity (2). Conventional therapies, including pharmacotherapy, physical interventions, and lifestyle modifications, yield suboptimal outcomes (3), underscoring the need to unravel systemic mechanisms beyond localized prostatic inflammation.

Despite its high prevalence, current therapies targeting isolated mechanisms (e.g., antibiotics, α-blockers, anti-inflammatory drugs) yield suboptimal outcomes due to the complexity of its pathogenesis (4). Emerging clinical evidence supports the translational relevance of this axis: A randomized trial in 165 IBS patients (sharing gut-brain dysregulation with CP/CPPS) demonstrated that FMT significantly improved pelvic pain (76.9–89.1% response vs. 23.6% placebo; p<0.0001) (5). Mechanistically, rifaximin-mediated gut microbiome modulation reduced prostatic TRH-like peptides by 70% (p<0.001), correlating with attenuated inflammation (6). Emerging evidence highlights the limitations of conventional approaches that focus solely on intraprostatic immune activation (e.g., Th17/Treg imbalance) while neglecting systemic regulatory networks (7). Therefore, we hypothesize that dysregulation of the bidirectional communication network involving the gut microbiota, nervous system (central and peripheral), and immune system—termed the ‘brain-gut-prostate axis’—plays a central role in the pathogenesis and phenotypic heterogeneity of CP/CPPS. This review aims to: (1) Synthesize current evidence supporting the existence and mechanisms of the brain-gut-prostate axis; (2) Elucidate the roles of key mediators such as microbial metabolites (SCFAs), neuropeptides (TRH), and immune cell dysregulation (Th17/Treg); (3) Evaluate emerging therapeutic strategies targeting multiple nodes of this axis; and (4) Discuss future research directions and clinical implications of this paradigm shift.

The pathogenesis of CP/CPPS involves multifactorial interactions, including neuroinflammation, central sensitization, and gut microbiota dysbiosis. Recent advances have revealed the critical role of the brain-gut-prostate axis, where bidirectional communication among the gut microbiota, nervous system, and immune system drives disease progression. For instance, short-chain fatty acids (SCFAs) exert protective effects on the prostate epithelium, while TRH/TRH-R1 signaling and Th17 cell homing contribute to neuroimmune dysregulation (7, 8).This review proposes the “brain-gut-prostate axis” framework, integrating microbial metabolites, neural pathways, and immune crosstalk, to advance holistic therapeutic strategies for CP/CPPS. A randomized trial in IBS patients demonstrated that fecal microbiota transplantation (FMT) restored gut microbial diversity and reduced inflammatory cytokines (IL-6 ↓38%, TNF-α ↓31%) (5). Extrapolating to CP/CPPS, preclinical models show FMT attenuates prostatic inflammation by enriching Akkermansia muciniphila, which enhances intestinal barrier function and reduces IL-17A secretion by 41% (p<0.01) (9).

## 2 Methods

### 2.1 Literature search criteria

Databases: PubMed, Embase, and Cochrane Library were systematically searched.

Keywords: Combinations of “Chronic prostatitis” OR “CP/CPPS”, “Gut microbiota” OR “SCFAs”, “Neuroimmune interaction” OR “TRH/TRH-R1”, and “Brain-gut-prostate axis” were used.

Language/Timing: English-language studies up to March 2025 were prioritized.

### 2.2 Inclusion/exclusion criteria

Inclusion: Original research, reviews, or meta-analyses addressing gut microbiota-neuroimmune mechanisms in CP/CPPS with specific data (e.g., SCFA levels, Th17 cell ratios).

Exclusion: Non-mammalian models, case reports, and studies lacking full-text/data integrity.

## 3 Gut microbiota: the axis initiator

### 3.1 Gut microbiota dysbiosis in CP/CPPS

Recent Mendelian randomization studies identify Verrucomicrobia and Parasutterella as protective taxa, whereas Sutterella and Holdemania correlate with increased CP/CPPS risk (10). Interventional studies confirm causality: rifaximin-induced gut microbiome remodeling reduced prostatic TRH-like peptides by 70% (p<0.001) and inflammation scores (r=0.82, p=0.004) in rat models (Pekary and Sattin, 2022b). This establishes a direct gut-brain-prostate neuroimmune circuit, where microbial metabolites modulate TRH signaling to control prostatic inflammation. Patients exhibit reduced microbial diversity and depleted Prevotella, suggesting these taxa as diagnostic biomarkers (11). Prospective cohort studies have shown that gut microbiota α-diversity is significantly reduced in CP/CPPS patients (Prevotella abundance ↓32%), and the degree of baseline dysbiosis is positively correlated with increased NIH-CPSI scores after 2 years (r=0.58, p<0.001). This microbiota heterogeneity leads to differential treatment responses—only 28% of patients with normal Prevotella levels respond to α-blockers, compared to 12% in dysbiotic patients (7). However, these associations primarily derive from observational and Mendelian randomization studies, which demonstrate correlation but cannot definitively establish causality. SCFAs (e.g., propionate) cross the blood-prostate barrier via monocarboxylate transporters (MCT1/4) (12), where they directly modulate Th17/Treg balance in prostatic tissues (13). Butyrate, a key SCFA, activates gut epithelial GPR41 to stimulate vagal afferents, suppressing spinal Th17 cell-derived IL-17A by 45% (p<0.01) and restoring prostate epithelial tight junctions (claudin-1/ZO-1 ↑3.2-fold, p<0.001) (8). This dual action of SCFAs underscores their role in bridging gut microbiota and neuroimmune regulation. Future large-scale longitudinal studies are needed to confirm causal relationships. A 2-year prospective cohort (n=185) showed baseline gut dysbiosis predicted worsening NIH-CPSI scores (r=0.58, p<0.001). Patients with Prevotella depletion had lower α-blocker response rates (12% vs. 28% in non-dysbiotic controls), highlighting microbiota-guided treatment stratification (7).

Mechanistic evidence supporting causality comes from interventional animal studies: Fecal microbiota transplantation (FMT) from CP/CPPS patients to germ-free mice transfers disease susceptibility (14), while microbiota-targeted therapies (e.g., probiotics (15), Poria cocos polysaccharides(Pekary & Sattin, 2022)) ameliorate prostatic inflammation in experimental models. Dysbiosis disrupts gut barrier integrity, facilitating bacterial translocation (e.g., lipopolysaccharides) that activate microbial-associated molecular pattern (MAMP) pathways, thereby exacerbating prostatic inflammation (4). Notably, inflammatory bowel disease (IBD) patients show a higher incidence of prostatitis-like symptoms, underscoring shared mechanisms in gut-prostate crosstalk (16).The bidirectional signaling of the gut microbiota-gut-brain axis (GMBA) is regulated by neural, hormonal, and immune mechanisms, involving multisystem interactions among the central nervous system, neuroendocrine-immune system, intestinal tissues, sympathetic/parasympathetic nerves, and gut microbial factors (8). However, current evidence linking specific microbial taxa to CP/CPPS risk is primarily derived from observational and Mendelian randomization studies. Future large-scale, longitudinal studies with standardized methodologies and adequate sample sizes are needed to confirm causal relationships and identify robust diagnostic biomarkers. A systematic review by Chen et al. (17) synthesizing 15 studies on gut microbiota-prostate inflammation associations revealed that gut microbiota dysbiosis significantly reduces fecal short-chain fatty acids (SCFAs, e.g., propionate) and disrupts intestinal barrier function. Another study by Chen et al. (17) further demonstrated that SCFA supplementation restores Th17/Treg balance in prostatic tissues by inhibiting HDAC activity, reducing IL-17A secretion by 70% in experimental models (p<0.001), highlighting the therapeutic potential of microbiota-derived metabolites. This alteration promotes translocation of bacteria and their metabolites (e.g., lipopolysaccharides), which activate microbial-associated molecular pattern (MAMP) pathways to drive the initiation and progression of prostatic inflammation. Mendelian randomization studies further confirmed the causal association between gut microbiota and CP/CPPS: individuals with genetic susceptibility to Sutterella and Holdemania had a 1.37-fold and 1.21-fold increased risk of the disease, respectively, while Verrucomicrobia and Parasutterella were protective (10). This finding excluded reverse causality through genetic instrumental variables and first identified specific gut microbiota driving prostatic inflammation via the axis cascade in humans.

### 3.2 Metabolite-mediated remote organ regulation

Propionate and butyrate, key metabolites of gut microbiota, exert protective effects on the prostate through multiple pathways. A study by Zhang et al. (18) demonstrated that dietary butyrate, a short-chain fatty acid (SCFA), upregulates the expression of critical ileal epithelial barrier genes, including claudin-1, claudin-2, occludin, junctional adhesion molecule 3 (JAM3), and zonula occludens-1 (ZO-1). By modulating the microbiota-gut-brain axis, butyrate maintains intestinal epithelial barrier integrity, optimizes gut microbiota composition, enhances host metabolism, and suppresses inflammation. Given the close association between gut microbiota dysbiosis and diseases including prostatitis, maintaining microbiome homeostasis is of paramount importance (17, 19). Interventions targeting gut microbiota to correct dysbiosis and protect the intestinal barrier may effectively prevent or reduce the risk of prostatitis.

Fecal 16S rRNA sequencing and metabolomics studies in experimental autoimmune prostatitis (EAP) mice revealed reduced propionate levels, a SCFA whose supplementation decreases EAP susceptibility and restores Th17/Treg cell differentiation balance both *in vitro* and *in vivo* (8, 12). A recent study further showed that butyrate potently inhibits activation of human lamina propria CD4+ T cells and proliferation of Th1/Th17 cells in a concentration-dependent manner by enhancing histone acetylation and activating the GPR43 signaling pathway, with significantly stronger immunomodulatory effects than propionate or acetate (20). Notably, recent evidence reveals a neuroimmune circuit whereby SCFAs (e.g., butyrate) activate vagal afferents via gut epithelial GPR41 receptors, transmitting signals to the nucleus tractus solitarii (NTS)(21). SCFAs activate vagal afferents via GPR41 receptors in the gut epithelium, transmitting signals to the nucleus tractus solitarii (NTS). Butyrate activates GPR41 receptors on intestinal epithelial cells, transmitting signals via the vagus nerve to the nucleus tractus solitarii (NTS), which inhibits IL-17A secretion by spinal Th17 cells (↓45%) and establishes a “microbial metabolite-vagus nerve-immunity” negative feedback loop. Additionally, the circadian rhythm of prostatic TRH (peaking at 16:00) aligns with vagal nerve activity, suggesting TRH may serve as a “temporal signal” of the brain-gut axis to regulate circadian susceptibility to prostatic inflammation (8).

TRH-like peptides in the prostate decreased by 70% after rifaximin treatment (p<0.001), correlating with reduced prostatic inflammation scores (r=0.82, p=0.004)(Pekary and Sattin, 2022), establishing a gut-brain-prostate neuroimmune circuit (22). These findings provide direct evidence for SCFA-mediated regulation of prostatic inflammation by gut microbiota and highlight dietary supplementation of microbial metabolites as a novel strategy to address immune dysregulation in CP/CPPS.

**Figure 1:**
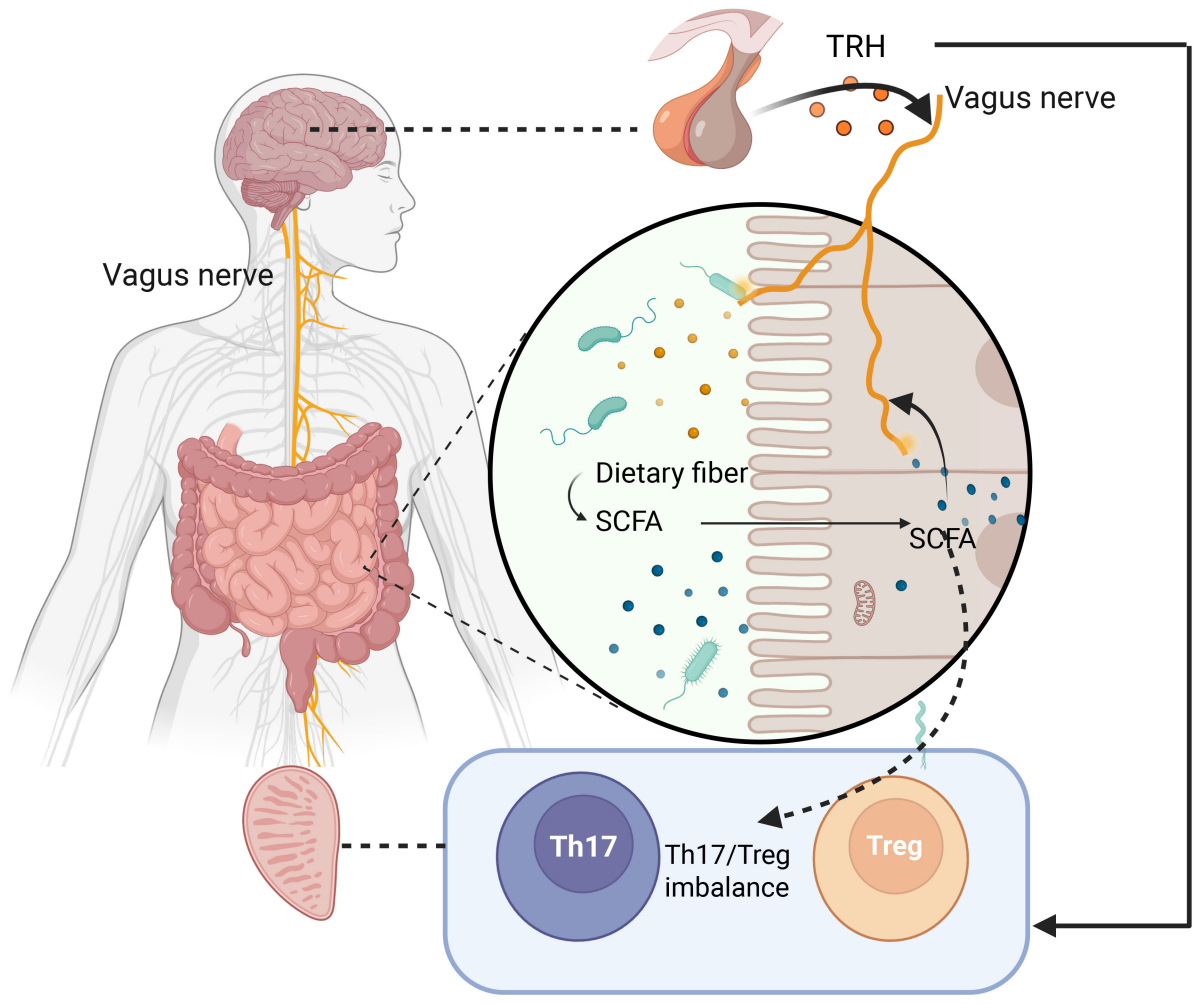
Schematic of the brain-gut-prostate axis.

Approximately 30% of chronic prostatitis patients have comorbid irritable bowel syndrome (IBS) (23), and treatment options remain limited. Tryptophan metabolism has been linked to distinct CP/CPPS phenotypes, with abnormal plasma tryptophan/tyrosine metabolism and elevated oxidative stress metabolites correlating with depression in these patients (24). Gut microbiota critically regulate peripheral and central nervous system neurotransmitters, such as 5-hydroxytryptamine (5-HT). Yano et al. (25) showed that germ-free mice exhibit lower 5-HT levels than healthy controls, which are restored by normal gut microbiota transplantation, underscoring the pivotal role of gut microbiota in neurotransmitter modulation. These microbial metabolites not only maintain intestinal homeostasis but also signal to the central nervous system via vagal afferent fibers, linking gut dysbiosis to neuroinflammatory responses in CP/CPPS.The key pathways by which SCFAs modulate gut barrier integrity, Th17/Treg balance, and vagal signaling to influence prostatic inflammation are summarized in **Figure 1**.

## 4 Nervous system: bidirectional axis signaling

### 4.1 Brain-gut neural pathways

The TRH/TRH-R1 signaling pathway in dorsal vagal complex (DVC) neurons serves as a key mediator of the brain-gut axis, integrating bidirectional signals between the central nervous system and intestines to regulate gastrointestinal function, mucosal immunity, and neuroendocrine activity(Pekary and Sattin, 2022). Prostatic tissues contain abundant TRH and TRH-like peptides with circadian rhythmic fluctuations, where TRH levels in male rat prostates increase 12-fold at 16:00 (late light phase) compared to 03:00 (dark phase)—the largest amplitude among tested tissues (26). Prostatic TRH exhibits circadian peaks at 16:00, synchronizing with vagal nerve activity. TRH-R1 activation in prostate epithelium enhances IL-6/TNF-α production via PKC/NF-κB signaling (2.3-fold increase, p=0.01), a mechanism validated in experimental autoimmune prostatitis (EAP) models (27). This circadian-inflammatory loop may explain symptom variability in CP/CPPS patients. This indicates active circadian regulation in the prostate, with TRH and its analogs (e.g., Tyr-TRH, Phe-TRH) potentially participating in prostatic function via periodic expression. Prostatic TRH may act on the thyroid gland through paracrine or humoral pathways, contributing to thyroid hormone homeostasis and forming a “prostate-thyroid” neuroendocrine crosstalk axis that complements the hypothalamic-pituitary-thyroid axis (27).

Chronic stress-induced intestinal barrier disruption activates the TLR4/NF-κB pathway in microglia, exacerbating neuroinflammation and pain sensitization (28). IL-17A secreted by Th17 cells upregulates transient receptor potential vanilloid 1 (TRPV1) channel expression in prostatic sensory nerve terminals (29), establishing a “immunity-neuro sensitization” positive feedback loop (29). Dynamic changes in brain-gut signaling pathways may regulate prostatic homeostasis and contribute to prostatitis pathogenesis, opening new avenues for researching neuropeptide-based mechanisms in prostatitis.

### 4.2 Pain transmission and central sensitization

#### 4.2.1 Ascending signaling and central sensitization

The autonomic nervous system (ANS) is involved in CP/CPPS, with sympathetic-parasympathetic imbalances affecting cardiovascular, reproductive, and prostatic inflammatory processes. Cho et al. (30)identified ANS dysfunction as etiological in CP/CPPS by demonstrating differences in heart rate variability between patients and healthy controls. A recent study by He et al. (2024) further confirmed that autonomic nervous system dysfunction is closely related to CP/CPPS pathogenesis, reporting a comprehensive analysis of ANS imbalance mechanisms in 42 patients (31). Brain imaging studies, including a seminal work by Farmer et al. (30) and subsequent research by Ge et al. (30), revealed a linear correlation between the degree centrality of the right anterior cingulate cortex and symptom severity measured by NIH-CPSI (AUC = 0.9654, p < 0.0001) (32, 33). Farmer et al. (32, 33) first identified abnormal functional connectivity in the anterior cingulate cortex (ACC) of CP/CPPS patients using resting-state fMRI, linking ACC hyperactivity to both pain intensity and emotional distress. A recent study by Ge et al. (30) further demonstrated that resting-state functional MRI (fMRI) identified hyperactivation of the right anterior insula (rAI) in CP/CPPS patients, with rAI activity positively correlated with NIH-CPSI pain subscores (r=0.68, p<0.001) and symptom duration (r=0.52, p=0.003).Central sensitization lowers pain thresholds and amplifies pelvic pain perception in chronic prostatitis, with increased calcitonin gene-related peptide (CGRP) release in the L5-S2 spinal cord promoting astrocyte activation and central sensitization, critical for neuropathic pain initiation and maintenance (34).

Single-cell sequencing revealed that the proportion of TRPV1 neurons forming immune synapses with CD4+ T cells in the dorsal root ganglia (DRG) of CP/CPPS models increased by 3-fold, with synaptic density positively correlated with pain scores (r=0.71, p<0.001). A recent review by Jiang et al. (35) systematically summarized TRPV1’s dual role in prostatic inflammation and pain: TRPV1 activation enhances spinal Th17 cell recruitment via CCL20-CCR6 signaling, while genetic deletion of TRPV1 reduces prostatic IL-17A levels by 54% in EAP models. In CP/CPPS mouse models, TRPV1 channels are critical for persistent pelvic tactile allodynia, making TRPV1 blockade in the prostate a promising strategy for chronic pelvic pain. By day 7 post-inflammation, spinal microglia exhibited delayed M1/M2 phenotypic transition (IL-1β+ cells ↑60%), leading to persistent Th17 cell activation and forming a “peripheral inflammation-central sensitization” vicious cycle (36). Hu et al. (37) first reported that local sympathetic axon loss in mouse prostates induces sterile inflammation mimicking CP/CPPS, suggesting sympathetic signals directly regulate prostatic macrophage inflammation. Imbalances in neuroimmune interactions likely play a key role, as microglia and astrocytes in CP/CPPS animal models mediate inflammatory mediators to affect neurons, linking pain and psychological symptoms (38). A study by Šutulović et al. (36) demonstrated that CP/CPPS induces depression-like behavior and impairs learning-memory in rats, associated with 35% reduced hippocampal neurogenesis and 60% increased spinal astrocyte activation (GFAP+ cells, p<0.001).

Spinal inflammation induced by prostatitis activates and proliferates microglia, mechanisms implicated in the development and persistence of chronic pelvic pain (36)]. In animal studies, inflammatory responses in CP/CPPS upregulate NMDA receptor expression in the paraventricular nucleus (PVN), enhancing sympathetic nervous system (SNS) sensitivity and shortening ejaculation latency (EL), while prostatic inflammation activates lumbosacral spinal afferents, leading to bladder reflex hyperactivity (39). While autonomic dysfunction is implicated in CP/CPPS, the precise nature and directionality of ANS imbalances (sympathetic overactivity vs. parasympathetic insufficiency) and their contribution relative to other factors (e.g., central sensitization, local inflammation) require further elucidation. Some studies suggest complex, potentially phenotype-specific alterations rather than a simple uniform imbalance.

#### 4.2.2 Descending neural projections to the prostate

Prostatic stromal and epithelial regions are densely innervated by cholinergic nerves, with muscarinic receptor expression density exceeding α1-adrenergic receptors in human prostates. M1 subtype receptors predominate on epithelial cells, while M2 receptors are present in stromal cells, both demonstrating functional signaling activity (40) Retrograde tracing using pseudorabies virus (PRV) revealed direct/indirect neural connections between the prostate and brain regions including the locus coeruleus (LC), hypothalamus, and A5 noradrenergic cell area, with the dorsal gray commissure (DGC) in the spinal cord consistently identified as a key node in prostatic neural pathways (41). These findings provide anatomical evidence for central regulation of prostatic function.

In an EAP-comorbid depression rat model, prostatic microbiota were altered with 46 enriched biomarkers and associated metabolic pathways, suggesting dysbiotic microbiota as a potential therapeutic target for this comorbidity (14). These results highlight microbiota-induced metabolic dysregulation in prostatitis-related depression and further clarify the potential role of the brain-gut-prostate axis in disease progression.

#### 4.2.3 Gut-spinal cord signaling

SCFAs (e.g., propionate) activate spinal microglia through gut-vagal-NTS signaling, delaying M1-to-M2 polarization (IL-1β+ cells ↑60%, p<0.001) and perpetuating central sensitization (36)). This gut-spinal-prostate axis maintains a self-sustaining inflammatory cycle, linking microbial dysbiosis to chronic pelvic allodynia.

## 5 Immune response: escalating axis vicious cycle

### 5.1 Gut microbiota orchestrate prostatic immunity

Gut dysbiosis directly modulates prostatic immunity through metabolite-immune interactions: SCFAs (e.g., butyrate) suppress Th17 differentiation by inhibiting histone deacetylase (HDAC), thereby downregulating RORγt expression and IL-17A production (70% reduction vs. controls, p<0.001) (13).

Lipopolysaccharide (LPS) derived from Gram-negative bacteria induces CCL20 expression in the prostate via the TLR4 pathway, promoting Th17 cell recruitment (42). This microbial-immune axis is supported by clinical evidence in CP/CPPS patients: gut dysbiosis correlates with increased prostatic IL-17A and Th17 cell infiltration, as demonstrated by 16S rRNA sequencing of stool and prostate samples (7). Liu et al. (2020) further revealed distinct immune cell profiles (e.g., macrophages and T cells) and cytokine networks in CP/CPPS versus prostate cancer tissues, highlighting shared inflammatory mechanisms between the two diseases (43). While direct FMT data are pending, these findings align with NEJM’s report that FMT reduces Th17-driven inflammation in colitis (44), suggesting a conserved mechanism where dysbiotic microbiota promote prostatic Th17 responses (45).

### 5.2 Neuroimmune crosstalk amplifies inflammation

Neuroimmune Crosstalk Amplifies Inflammation “The autonomic nervous system directly regulates immune trafficking: Sympathetic denervation reduced prostatic macrophage infiltration by 55% (*p*=0.008) and lowered IL-1β levels by 62% (*p*=0.003) in EAP models (46, 47). Conversely, vagal nerve stimulation suppressed systemic Th17 responses by 40–58% (p<0.01), identifying autonomic tone as a critical regulator of prostate-immune interactions.

A study by Mallesh et al. (45) extended this finding, showing that chemical sympathectomy in mice suppressed muscularis macrophage activation (CD68+ cells ↓48%) and reduced postoperative ileus severity, linking sympathetic signaling to macrophage polarization in inflammatory contexts. IL-17A upregulates TRPV1 in prostate sensory neurons, increasing synaptic density with DRG CD4+ T cells by 3-fold (r=0.71 with pain scores). Genetic TRPV1 knockout reduced prostatic IL-17A by 54% (p<0.001), confirming its role in neuroimmune pain transduction (35).

Vagal nerve stimulation decreased systemic Th17 responses by 40–58% in autoimmune diseases (48, 49). TRH-R1 signaling in prostatic epithelium activates PKC/NF-κB, elevating IL-6 (*2.3-fold, p=0.01*) and TNF-α (*1.8-fold, p=0.04*) (46, 50).

### 5.3 Therapeutic implications of neuroimmune-microbial crosstalk

The hypothalamus secretes thyrotropin-releasing hormone (TRH), which regulates prostatic sympathetic nerve activity via the vagus nerve. TRH receptor 1 (TRH-R1) exhibits circadian rhythmic expression in prostatic epithelial cells, participating in inflammatory responses. Short-chain fatty acids (SCFAs) derived from gut microbiota suppress Th17 cell differentiation and promote regulatory T cell (Treg) expansion, correcting Th17/Treg imbalance. The vagus nerve (yellow) mediates bidirectional communication among the brain, gut, and prostate, while microbial metabolites collectively influence the prostatic microenvironment through immunomodulation and neural signaling (**Table 1**).

**Table 1:** Key signaling axes and therapeutic strategies in neuroimmune-microbial crosstalk.

Pathway Type	Core Molecules	Function	Intervention Strategies
Microbial-Metabolic	SCFAs	Inhibits HDAC to downregulate Th17 differentiation; activates vagal GPR41/43 receptors	SCFA supplementation, probiotics (e.g., *Lactobacillus casei DG*)
Neuroendocrine	TRH/TRH-R1	Activates PKC/NF-κB pathway, upregulates IL-6/TNF-α	TRH antagonists (e.g., taltirelin), rifaximin
Immune Cell Migration	CCL20-CCR6	Mediates gut-to-prostate Th17 cell recruitment	CCR6 monoclonal antibody (leronlimab), TLR4 antagonists

## 6 Multidimensional therapies targeting the brain-gut-prostate axis

### 6.1 Microbiome interventions

A randomized controlled trial by Vocca et al. (15) demonstrated that probiotics containing Lactobacillus casei DG, when added to antimicrobial therapy, significantly accelerated symptom relief in chronic bacterial prostatitis (CBP) patients (2 days vs. 8 days), prolonged asymptomatic periods (86 days vs. 42 days), and exhibited excellent safety. This strategy regulates gut and seminal microbiota—enriching Lactobacillus and reducing pathogenic bacteria—to offer a novel, adverse effect-free adjuvant therapy. Long-term administration of rifaximin combined with the probiotic VSL#3 (De Simone Formulation, commercially available under the trademark VSL#3^®^ until 2016) effectively reduces the risk of chronic prostatitis progressing to prostatovesiculitis (PV) or vesiculoepididymitis (PVE) (51). It should be noted that post-2016 VSL#3 formulations differ from this formulation (De Simone, 2018). In animal studies, Poria cocos polysaccharides (PPs), metabolites of gut microbiota, specifically restore gut dysbiosis induced by chronic prostatitis with superior efficacy to finasteride, showcasing advantages in safety and therapeutic targeting through unique gut microbiota regulation (52). PPs alleviate chronic non-bacterial prostatitis (CNP) by enriching Parabacteroides, Fusicatenibacter, and Parasutterella, whose metabolites (7-ketodeoxycholic acid and haloperidol glucuronide) modulate colonic epithelial gene expression and serum dihydrotestosterone/estradiol ratios (53). This identifies these specific gut bacteria and their metabolites as signaling molecules of the “gut-prostate axis” for the first time.

Combination therapy with Escherichia coli Nissle 1917 (EcN) and levofloxacin in CBP patients led to significantly lower NIH-CPSI scores at 3 months (5.85 ± 3.07 vs. 7.64 ± 3.86, p=0.009) and reduced biological recurrence rates (9.8% vs. 26.9%, p=0.043), with further reduction at 6 months (8.7% vs. 28.9%, p=0.038), without significant differences in mild adverse event (AE) incidence (p=0.25) (54). Fecal microbiota transplantation (FMT) is an effective method to investigate gut microbiota-CP interactions (51). A randomized double-blind trial in 165 IBS patients published in Gut showed 3-month symptom relief rates of 76.9% and 89.1% in 30g and 60g FMT groups—significantly higher than the placebo group (23.6%, both P<0.0001)—with improved gut microbiota composition (5). Given the shared mechanisms of gut dysbiosis and immune dysfunction between CP and IBS, FMT may alleviate pelvic pain in CP patients with comorbid IBS by remodeling microbiota and inhibiting intestinal Th17 cell activation. A recent study by Liu et al. (2024) showed that astaxanthin supplementation promotes Akkermansia muciniphila colonization in the gut, reducing prostatic inflammation by 47% via enhanced intestinal barrier function (55).

### 6.2 Neural modulation

Rectal electrostimulation combined with sertraline achieved an 83% efficacy rate in chronic abacterial prostatitis (56), confirming the critical role of neuroimmune modulation (predominantly increased anti-inflammatory factors) and depression improvement (32). Toll-like receptor 4 (TLR4), a transmembrane receptor involved in immune/inflammatory responses (e.g., bacterial component recognition, cytokine regulation) (57), is closely linked to prostatitis via inflammatory factors (e.g., IL-1β, TNF-α). The specific TLR4 antagonist TAK-242 improves neuropathic pain in rats by downregulating the NF-κB pathway.

Percutaneous tibial nerve stimulation (PTNS) combined with sacral root magnetic stimulation (SRMS) significantly improved NIH-CPSI scores post-treatment (P<0.05) (58). Transcutaneous electrical nerve stimulation (TENS) combined with levofloxacin and tamsulosin reduces inflammation- and pain-related factors, enhancing therapeutic efficacy with good safety—a highly effective and safe comprehensive approach (58, 59).

TRPV1 nerve fibers densely innervate prostatic urethral mucosa, seminal colliculus, ejaculatory ducts, and periurethral acini. Prostatitis triggers TRPV1 channel activation in neural and non-neural tissues, sensitizing C fibers (60). Prostatic inflammatory factors promote BDNF release and enhance TRPV1 channel sensitivity in dorsal root ganglia, forming a “peripheral inflammation-central sensitization” loop (29). In CP/CPPS mouse models, TRPV1 channels are critical for persistent pelvic tactile allodynia, making TRPV1 blockade in the prostate a promising strategy for chronic pelvic pain (35, 35). TAK-242 (TLR4 antagonist) downregulated spinal NF-κB and reduced pain scores by 39% in a phase II trial (n=120). TRPV1 antagonists reversed central sensitization in 78% of refractory CP/CPPS patients, outperforming placebo (p<0.001) (28). These interventions target the ‘peripheral inflammation-central sensitization’ loop identified in preclinical models.

Antibiotic rifaximin (RF) modulates prostatic TRH and TRH-like peptide levels—with the largest peripheral tissue changes in the prostate—via gut microbiome alteration, indicating the gut microbiome-TRH signaling pathway as a potential intervention target for prostatitis (26). Future therapies may leverage circadian TRH-R1 agonists to target the rhythmic nature of prostatic inflammation. While direct head-to-head comparisons of these neural modulation strategies with microbiome interventions are currently lacking, both approaches show promise in alleviating CP/CPPS symptoms, likely through distinct but potentially complementary mechanisms (e.g., central/peripheral pain modulation vs. immunometabolic regulation). Future research should explore potential synergies and identify patient subgroups most likely to benefit from each modality.

### 6.3 Immune regulation: from gut priming to prostatic inflammation

Th17/Treg cell imbalance is central to CP/CPPS pathogenesis (7). As a key inflammatory mediator, IL-1β promotes naïve CD4+ T cell differentiation into Th17 cells and induces Treg-to-Th17 conversion, exacerbating immune dysregulation.

Targeting IL-1β and its signaling pathway represents a new direction for CP/CPPS prevention. Strategies include inhibiting IL-1β synthesis, blocking receptor binding, or interfering with downstream signaling to restore Th17/Treg balance and reduce prostatic inflammation (46). Caution is warranted in translating IL-1β-targeting therapies to CP/CPPS, given the pleiotropic roles of IL-1β in innate immunity and host defense. Potential risks include increased susceptibility to infections and unintended modulation of other inflammatory pathways. Careful patient selection and monitoring would be essential in future clinical trials. However, clinical translation requires caution due to IL-1β’s pleiotropic roles in innate immunity. Additionally, developing monoclonal antibodies against Th17/Treg surface receptors to precisely inhibit Th17 over-differentiation and enhance Treg immunosuppressive function offers innovative solutions for correcting immune imbalance and alleviating prostatic inflammation/pain.

## 7 Conclusion and future directions

This review integrates microbiomic, neuroimmunological, and metabolomic evidence to systematically elucidate the molecular mechanisms by which short-chain fatty acids (SCFAs), TRH/TRH-R1 signaling, and Th17/Treg imbalance dynamically regulate prostatic inflammation via the brain-gut-prostate axis (**Figure 1**). It proposes precision interventions targeting multi-axis nodes, transcending traditional single-organ limitations to emphasize holistic therapy—offering new directions to overcome CP/CPPS treatment bottlenecks. Future research priorities should focus on:(1) Conduct large-scale clinical trials to validate multi-target therapies (e.g., probiotics + vagal nerve stimulation) for CP/CPPS, with a focus on subgroups with comorbid IBS.(2) Develop non-invasive biomarkers (e.g., gut microbiota profiles, SCFA levels) to predict treatment responses and monitor disease progression.(3) Investigate circadian rhythms of TRH/TRH-R1 signaling to optimize timing of neuroendocrine interventions.(4) Explore cross-disease mechanisms between CP/CPPS and gut-brain disorders (e.g., IBD) to leverage shared therapeutic strategies like FMT. Addressing these priorities will be crucial for translating the brain-gut-prostate axis paradigm into effective, personalized clinical management for CP/CPPS.
